# A long distance telesurgical demonstration on robotic surgery phantoms over 5G

**DOI:** 10.1007/s11548-023-02913-2

**Published:** 2023-04-24

**Authors:** George Moustris, Costas Tzafestas, Konstantinos Konstantinidis

**Affiliations:** 1 School of Electrical and Computer Engineering, National Technical University of Athens, Zographou Campus, 15773 Athens, Greece; 2 Department of General, Bariatric, Laparoscopic and Robotic Surgery, Athens Medical Centre, Athens, Greece

**Keywords:** Robotic surgery, Telesurgery, 5 G, Remote surgery, Surgical phantoms

## Abstract

**Purpose:**

Using robotic technology and communications infrastructure to remotely perform surgery has been a persistent goal in medical research in the past three decades. The recent deployment of the Fifth-Generation Wireless Networks has revitalized the research efforts in the telesurgery paradigm. Offering low latency and high bandwidth communication, they are well suited for applications that require real-time data transmission and can allow smoother communication between surgeon and patient, making it possible to remotely perform complex surgeries. In this paper, we investigate the effects of the 5 G network on surgical performance during a telesurgical demonstration where the surgeon and the robot are separated by nearly 300 km.

**Methods:**

The surgeon performed surgical exercises on a robotic surgery training phantom using a novel telesurgical platform. The master controllers were connected to the local site on a 5 G network, teleoperating the robot remotely in a hospital. A video feed of the remote site was also streamed. The surgeon performed various tasks on the phantom such as cutting, dissection, pick-and-place and ring tower transfer. To assess the usefulness, usability and image quality of the system, the surgeon was subsequently interviewed using three structured questionnaires.

**Results:**

All tasks were completed successfully. The low latency and high bandwidth of the network resulted into a latency of 18 ms for the motion commands while the video delay was about 350 ms. This enabled the surgeon to operate smoothly with a high-definition video from about 300 km away. The surgeon viewed the system’s usability in a neutral to positive way while the video image was rated as of good quality.

**Conclusion:**

5 G networks provide significant advancement in the field of telecommunications, offering faster speeds and lower latency than previous generations of wireless technology. They can serve as an enabling technology for telesurgery and further advance its application and adoption.

**Supplementary Information:**

The online version contains supplementary material available at 10.1007/s11548-023-02913-2.

## Introduction

Due to its significant value, the projection of surgical skill over long distances through electromechanical means, i.e., remote surgery, has been a consistent goal in surgery since the advent of surgical robots. Indeed, one of its main advantages is the ability to perform surgery on patients who are located in remote or underserved areas, which could significantly increase access to medical care for individuals in those areas and reduce the need for patients to travel long distances, to receive treatment. Furthermore, surgeons can remotely perform surgical procedures on patients who may not have access to specialized medical facilities or trained medical professionals in their local area. This could be particularly beneficial in developing countries, where access to quality medical care is often limited, or even in extreme environments such as space and under the sea [1]. In addition, telesurgery has the potential to reduce the cost of medical care by eliminating the need for patients to physically travel to a hospital or clinic as well as reduce the workload on surgeons, since they would be able to perform multiple surgeries remotely rather than being physically present for each procedure.

Following the pioneering transatlantic *Lindbergh Operation* from New York to Strasbourg, performed by Prof. Marescaux in 2001 [2, 3], over the past two decades there have been several demonstrations of surgeons operating remotely with robotic instruments to perform various interventional tasks. Prominent examples include the telesurgery program established in Canada by Prof. Anvari [4, 5], the NEEMO missions for underwater medical interventions [6, 7], remote surgery experiments between Japan and Thailand over the Internet [8], field deployable surgical robots using Unmanned Aerial Vehicles, to provide airborne wireless communications links [9, 10] and others (for a more thorough exposition of the history and experiments in telesurgery, we refer the reader to [11–13]).

Crucial components of remote surgery are the perceived *latency* in the communication between the surgeon and the robot [14–16], concerning both the issued motion commands and the video streamed back from the robot to the surgeon, as well as the *quality* of the video image itself [17, 18]. Research has shown that latency in the range of 0–200 ms is ideal for telesurgery with most surgeons barely noticing that something is off. Deterioration of skill is observed at a latency $\ge$ 300 ms, and the errors increase from 500 ms and above [19]. A delay greater than 700 ms is deemed unsuitable for telesurgery. Therefore, a latency up to 300 ms is considered safe with 400–500 ms being also acceptable, but tiring. The emergence of 5 G technology has the potential to significantly enhance the capabilities of telesurgery. 5 G networks offer much faster speeds and lower latency than previous generations of wireless technology, making them well-suited for applications that require real-time data transmission. This makes 5 G an attractive option for telesurgery as it allows surgeons to remotely control surgical instruments with greater precision and responsiveness.

In this paper, we report on a set of telesurgery experiments over 5 G in Greece using a novel prototype surgical robot called the “Double Delta” [20]. The experiments consisted of simple surgical exercises on a robotic surgery training kit, viz. the Fundamentals of Robotic Surgery (FRS) Dome [21]. The surgeon was located at the city of Trikala, Thessaly Greece, teleoperating the robot some 300 km away in an operating room at the Athens Medical Center in Marousi, Athens. In the following, we present the setup of the system, the communications infrastructure, various tasks performed, and the qualitative assessment of the usability and image quality of the platform.

## Related works

In recent years, there has been an increasing interest in utilizing 5 G technology to enhance the remote surgery paradigm, with demonstrated interventions steadily emerging. Acemoglu et al. [22] presented a remote transoral laser microsurgery procedure on an adult human cadaver. The surgeon used a tablet computer to position the laser and a haptic device to control the surgical forceps. The local and remote sites were separated by a distance of 15 km.

The authors in [23] report on the first twelve cases of telerobotic spinal surgery over 5 G. They present a “one-to-many” paradigm where a surgeon in a master control room operates at the same time on many remote sites. The master control room was located in Beijing, China, while the patients who underwent operations were placed in hospitals in various cities across the country. The average latency was 28 ms with no network adverse events.

The application of ultra-remote robot-assisted laparoscopic surgery in China using 5 G, was presented in [24]. The authors described four successful operations (left nephrectomy, partial hepatectomy, cholecystectomy and cystectomy) performed on a swine model. The master controller and the remote site were connected through a Virtual Private Network over 5 G, separated by nearly 3000 km. Total reported average delay, including network latency, image capturing, video (de)coding and mechanical response of the robot, was 264 ms. Recently, the same team presented an ultra-remote telesurgical robot-assisted laparoscopic radical cystectomy on a human patient [25].

Robotic telestenting has also been demonstrated in [26]. The authors performed regional (from Boston to New York, $\sim$ 300 km) and transcontinental (from Boston to San Francisco, $\sim$ 4970 km) experiments using both a landline and a 5 G connection. The surgeon operated a master console located at a Hospital in Boston, whereas in the remote sites an endovascular simulator was deployed, using an endovascular robotic system to perform the coronary interventions. In the regional trials, 20 attempts were performed in various target lesions (10 over wired and 10 over 5 G connections). Similarly, 16 attempts were carried out in the transcontinental case (9 wired, 7 5 G). All procedures were successful. Reported latency was 121.5 ms vs 162.5 ms for the wired vs 5 G links in the transcontinental case, and 67.8 ms vs 86.6 ms in the regional case, respectively. The authors concluded that there weren’t any significant differences in the performance of the telestenting procedures with regard to distance.

Besides telesurgical procedures, 5 G has also been used to transmit physiological and imaging information. For example, in [27] the authors transmitted in real time anonymized perinatal data (cardiotocogram, HD ultrasound, HD video) from actual normal fetuses. The observed latency was less than one second with no apparent loss of quality. The local and remote sites, however, were located on the same floor of the premises.

A residential telemonitoring system which collects physiological and environmental data, streaming them over 5 G, is analyzed in [28]. The system captured signals from a respiratory motion tracker, a pulse oximeter and an environmental sensor that measures temperature, pressure, relative humidity, etc. It was designed to continuously identify the activity and the respiratory parameters of the monitored subjects. The data were sent to a central database via a local 5 G router and analyzed in real time. The trials involved 18 healthy subjects who wore the device for at least 48 h. The system performed unsupervised during the experiments, providing real-time monitoring of the volunteers.

5G-assisted telementored surgery is mentioned in [29]. The authors present two cases of laparoscopic procedures where the endoscopic image, along with audio, was streamed from an OR to an auditorium in Barcelona ($\sim$ 4 km away), in the first case, and in Shanghai ($\sim$ 6 km away) in the second. Reported latency was 202 ms and 146 ms, respectively, with the image quality being favorably rated by the surgeons.

Remote ultrasonography has been demonstrated in several cases. For example, emergency mobile bidirectional communication between an ambulance and a hospital has been described in [30]. Ultrasound images, audio and video from the ambulance were transmitted in real-time to the command center using 5 G. Average latency was 10 ms with a good image quality. Furthermore, in response to the COVID-19 pandemic, remote robotic ultrasound for patient examination has been been deployed on the field in China [31–33]. Remote lung ultrasonography using 5 G was tested as a means to provide more immediate consultations and physical separation between patients and medical personnel. For a more in-depth discussion regarding recent efforts concerning the application of 5 G in healthcare, the reader is referred to [34].

## Materials and methods

### Location and networking

The telesurgery demonstration was a collaboration between the National Technical University of Athens, the Athens Medical Centre and Vodafone Greece. The aim was to observe the performance of the 5 G communications network with respect to a demanding use case such a remote surgery, to investigate the effects of ultra-low latency and high bandwidth on remote surgical manipulation and also to provide a first demonstration of the novel Double Delta surgical robot in a relevant environment.

**Fig. 1:**
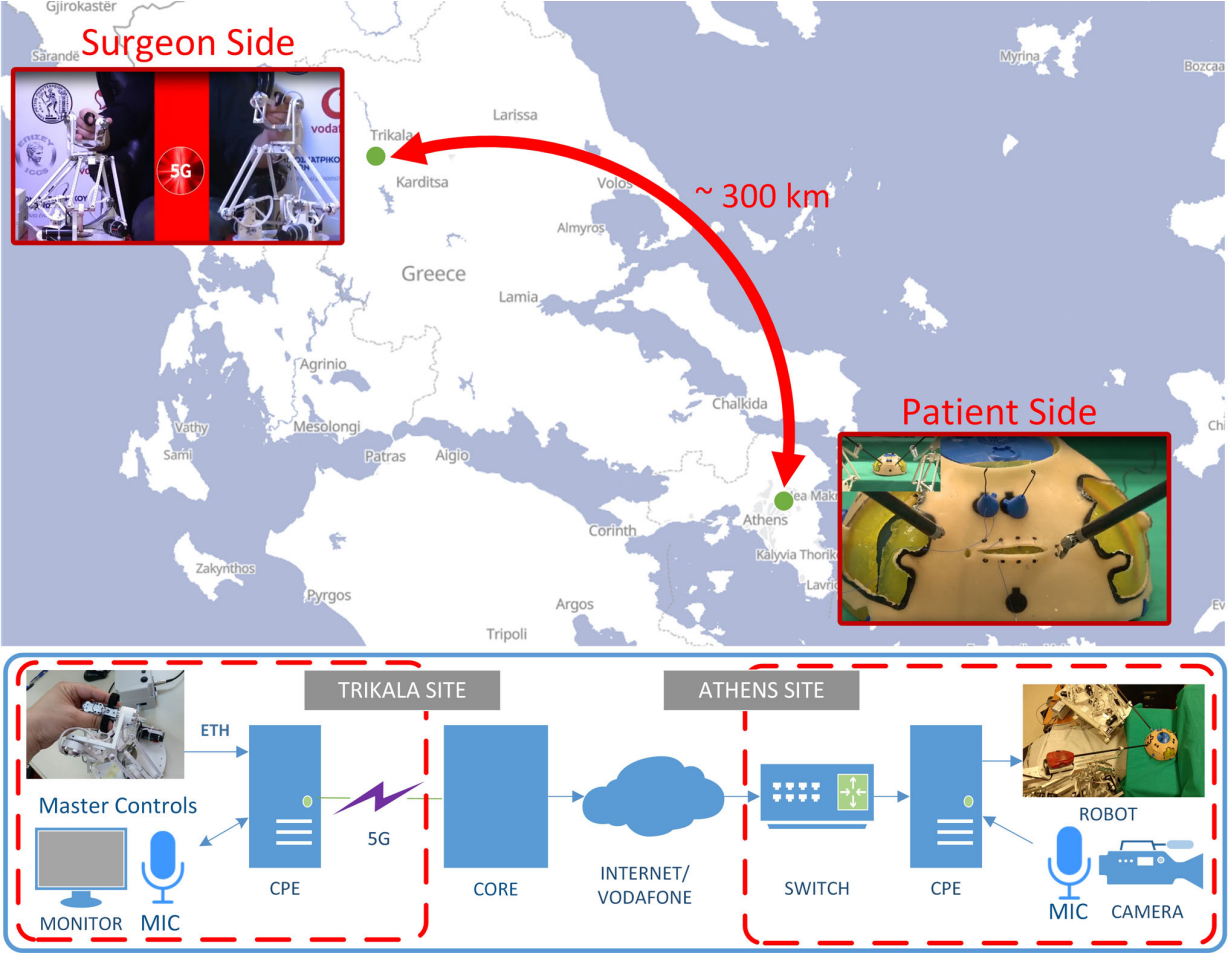
(Up) Presentation of the geographical locations of the two sites of the telesurgery demonstration. (Down) Abstract network diagram of various connected components at the two locations

The surgeon console, comprising the master controls, imaging equipment for viewing the remote site and telecommunications equipment, was located at a suitable hotel area at Trikala (Fig. 1). A portable 5 G antenna was transferred to the demo area, providing local 5 G coverage. Speed tests measured its download (DL) throughput to 800 Mbps$-$1.4 Gbps and its upload (UL) throughput to 60–70 Mbps. The telesurgical equipment was connected to a 5 G router (Huawei 5 G CPE Pro 1) through a wired Gigabit Ethernet connection (ETH). The CPE provided 1 Gbps DL/ 250 Mbps UL speeds to the 5 G antenna.

The remote site was located at an operating room inside the Athens Medical Centre hospital, where the slave robot was residing, along with networking and video equipment. The telesurgical equipment was connected to the hospital’s local wired Gigabit Ethernet network through a switch. This was routed to the Internet via Vodafone’s Fixed Network, enabling communication with the Trikala site via a UDP connection.

### Robotic equipment

**Fig. 2:**
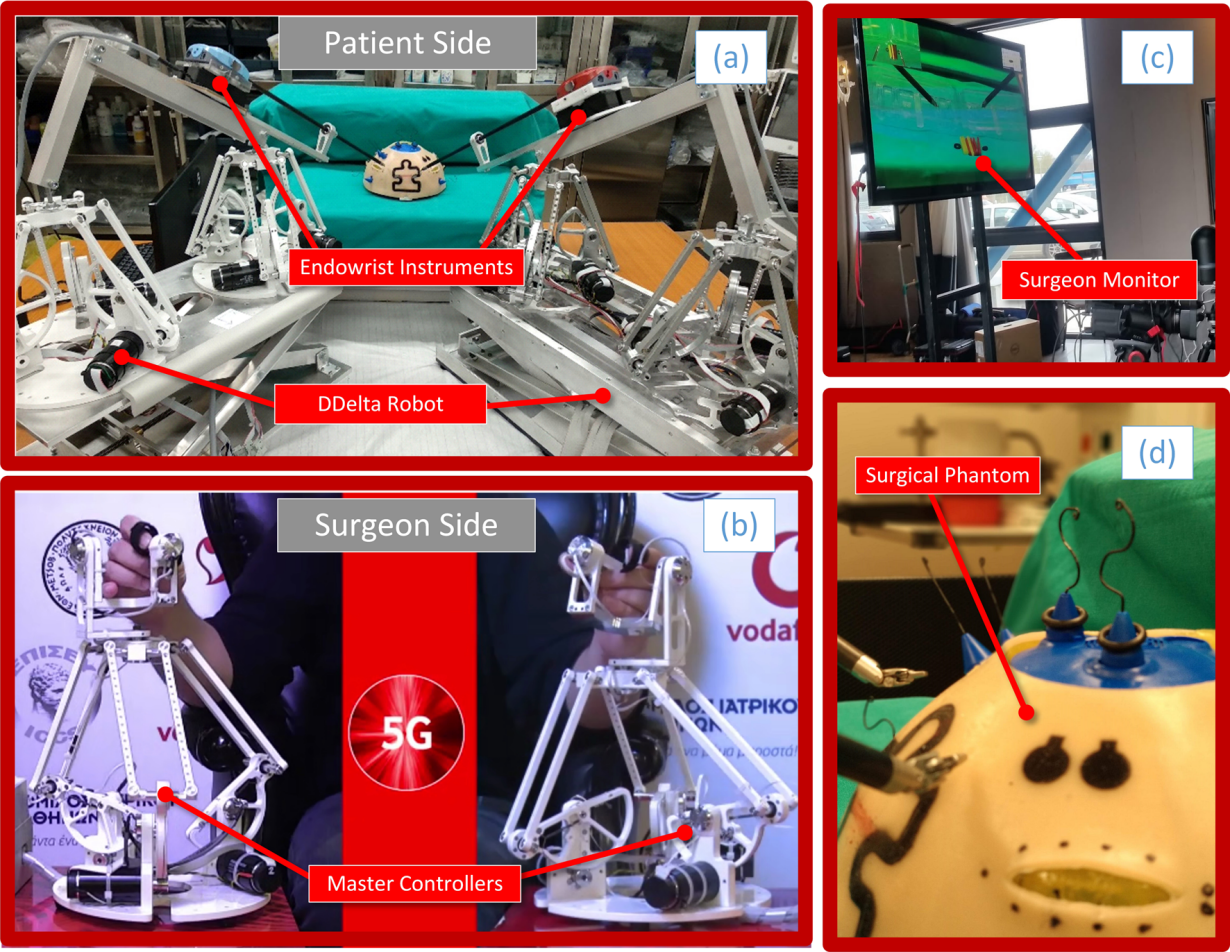
**a** View of the patient side. The DDelta robot is visible, bearing two endowrist instruments. **b** View of the surgeon’s master controllers at the surgeon side. **c** Surgeon’s monitor depicting the patient side. **d** The FRS Dome used as a training phantom

A secondary aim of the telesurgical demonstration was to validate the novel robotic platform used for the experiments. The Double Delta, or simply *DDelta* (Fig. 2a), is a new research-oriented master–slave telesurgical robot, based on parallel kinematics and was developed by the National Technical University of Athens, in collaboration with the Lublin-based robotics company Accrea Engineering (Lublin, Poland). The master manipulator is based on the parallel configuration of two Delta robots, thus capitalizing on the widely available research literature on their modeling, analysis and control. The manipulators are interfaced with da Vinci’s Endowrist robotic surgical instruments. Key advantages of the parallel design are the simpler inverse kinematic solutions, the higher accuracy and speed, the lighter weight and the enhanced rigidity of the structure. Furthermore, due to the parallel actuation, errors inserted into the kinematic chain are meliorated, leading to a lesser impact on precision. A drawback, however, is the often smaller workspace and more complex forward kinematics. Preliminary details on the design are provided in [20], although the platform used in this demonstration was a second iteration of the design.

The master controllers are also based on the concept of the Delta robot, providing 7 Degrees-of-Freedom (DoF) sensing (3 rotations, 3 translations, 1 pinch) and 3 Dofs actuation (Fig. 2b). They are thus haptic-enabled and can project forces on the surgeon’s hand. The controllers are connected to the local 5 G CPE and are coordinated by a local laptop computer. This computer establishes the UDP connection to the remote site, sending the motion commands. It should be noted that, at this time, the Double Delta platform is a prototype, built as a research tool and is not intended for use on actual patients.

### Video pipeline

The surgeon viewed the robot using video from a monocular camera located at the remote site. This image was transmitted over the internet and displayed on a large overhead monitor at the local site (Fig. 2c). The camera used was a SONY NX5R, producing a 1080i50 10bit 4:2:2 uncompressed video feed through a 3 G SDI output. This was captured by a video grabber card (Blackmagic DeckLink DUO 2 mini) on a local workstation and was compressed to 1080p25 using the H264 codec, with a variable bitrate of 1.5–2 Mbps. This was performed using hardware encoding with the NVIDIA Encoder (NVENC) on a QUADRO P620 graphics card. The feed was then streamed over the internet to the surgeon’s site using a commercial video streaming service (Zoom). The required bandwidth was 2Mbps. The average latency to the Zoom server was 25 ms. Overall end-to-end latency, from the patient side to the surgeon side, was 250 ms on average.

### Surgical exercises

**Fig. 3:**
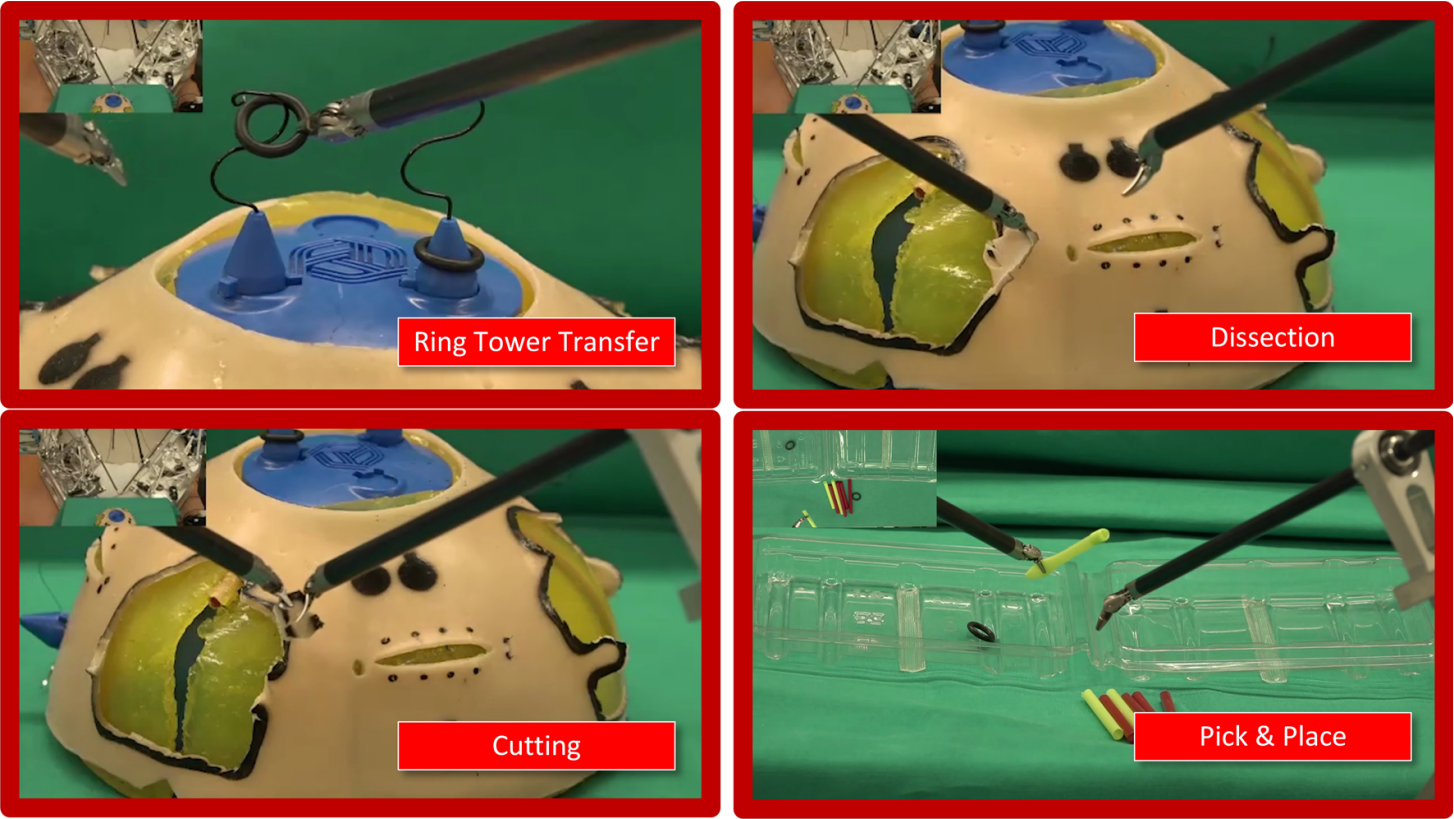
Snapshots of the four exercises performed during the telesurgery demonstration. The first three (ring transfer, dissection, cutting) were performed on the FRS Dome while the fourth was a custom pick and place board

During the two days of the demonstration, the surgeon performed a number of trials in various training exercises using a standardized phantom for robotic surgery training, viz. the FRS Dome [35] (Fig. 2d). The Dome is part of a psychomotor skills curriculum, designed to train and assess the proficiency of surgeons who are interested in performing robotic surgery. The curriculum was developed through multiple consensus conferences, which brought together subject matter experts from multiple surgical societies, surgical educational societies, surgical boards and other governing organizations [21]. The FRS Dome contains seven tasks; Docking and Instrument Insertion, Ring Tower Transfer, Knot Tying, Railroad Track, 4th Arm Cutting, Puzzle Piece Dissection and Vessel Energy Dissection. Out of the seven, the following three were selected: Ring Tower Transfer, Dissection and Cutting (see Fig. 3 for snapshots of the trials). A fourth one, a pick and place exercise, was also added using rubber rings and tubular plastic pieces.

### Subjective evaluation criteria

For the subjective evaluation, after the demonstration the surgeon was presented with three structured questionnaires regarding the assessment of the robot usability and the image quality. For the former, we used the *Modified System Usability Scale* and the *Robot Usability Score* found in [36], deployed in a similar telesurgical evaluation experiment. For the latter, the *Video Quality Assessment* questionnaire in [18] was used. Finally, the surgeon was also asked to optionally provide any other free-text general remarks about the demonstration.

The **Modified System Usability Scale** (m-SUS) is based on the *System Usability Scale* which was developed as a reliable tool for measuring the usability of systems. The m-SUS was used to evaluate the usability of the telesurgical system in our demonstration. It consists of nine questions rated in a 5-point Likert scale, in descending order of usefulness (see Fig. 5 in Appendix). The score for each individual answer was collected, and the total score was then calculated.

The **Robot Usability Score** (RUS) was created by the authors in [36], used to evaluate the usability and usefulness of the robotic system. It evaluates eight items: physical comfort, manual operability, foot pedal operability, stereoscopic performance, forceps operability, smoothness, satisfaction and effectiveness. Answers are given in a 5-point Likert scale in descending order of usefulness (see Fig. 6 in Appendix).

The **Video Quality Assessment** questionnaire consists of five questions which investigate the relevant aspects of the video: colors, edges, details, 3D relief and so on (see Fig. 7). It uses a discrete scale from “0” (bad) to “10” (excellent) and was also used for the same purpose in [18].

## Results

During the exercises, the measured round-trip time (i.e., ping) from the surgeon’s controllers to the robot at the remote site, and back, was 18 ms. Video transmission from the remote site to the network end on the surgeon’s site was 250 ms. A further 100 ms was added from there to the surgeon’s monitor, thus resulting in 350 ms latency for the entire video pipeline (from the camera at the patient side to the monitor at the surgeon’s site).

**Fig. 4:**
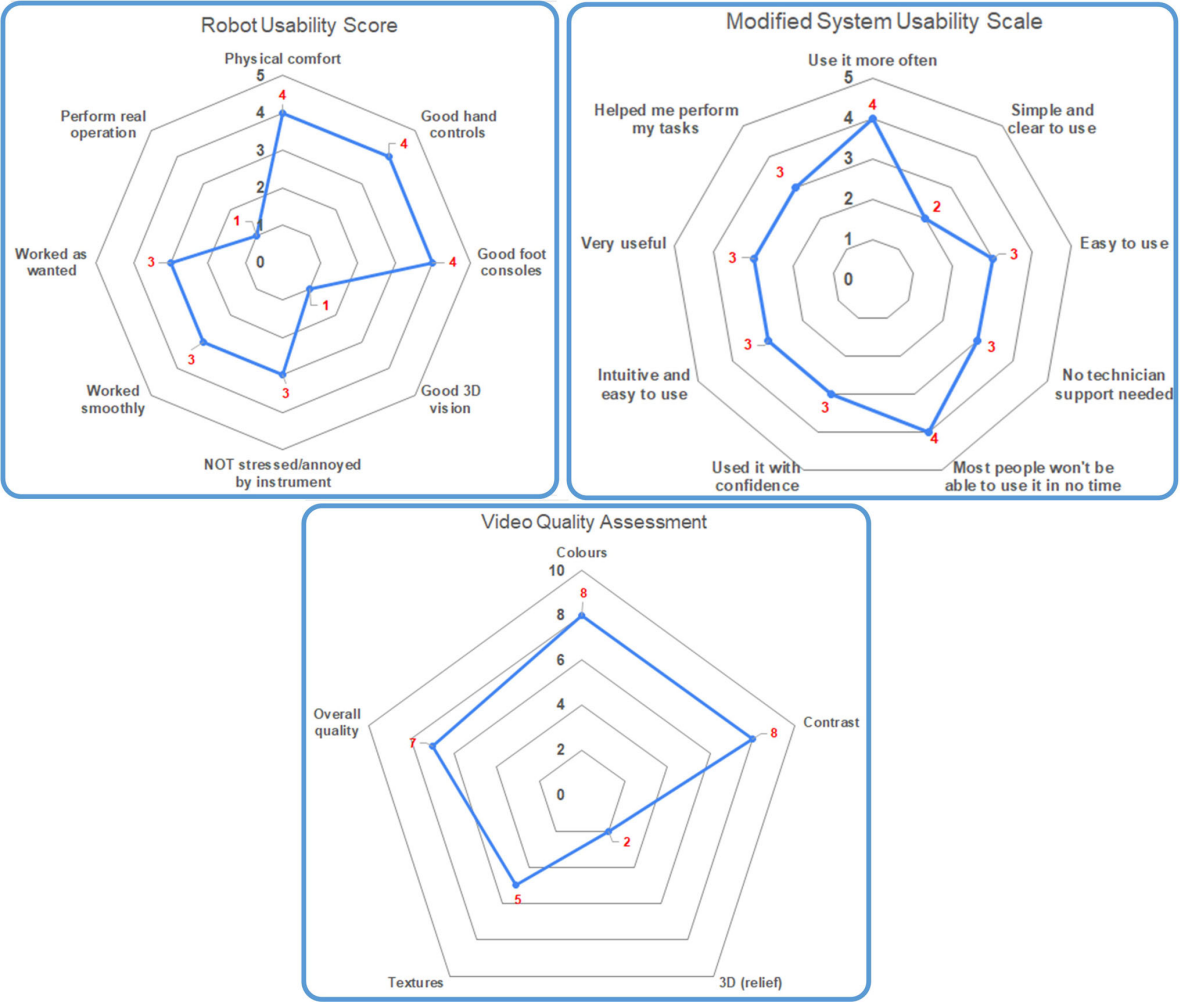
Spider graphs of the answers from the three questionnaires; m-SUS scale is 1 = strongly disagree, 5 = strongly agree; RUS scale is 1 = strongly disagree, 5 = strongly agree; Video Quality Assessment scale is 0 = bad, 10 = excellent

The total score for the** m-SUS** was *28*, with the individual question scores being presented in Fig. 4. Specifically, the surgeon responded that he would like to have more sessions with the system (“use it more often” = 4), but the robot was difficult to use (“Simple and clear to use” = 2, “Easy to use” = 3). Support from appropriate technical personnel might also be good to have (“No technician support needed” = 3) since most people might find it hard to operate initially (“Most people won’t be able to use it in no time” = 4). Furthermore, he operated with relative confidence (“Used it with confidence” = 3) and the robot’s controls behaved largely as expected but seemed manneristic at times (“Intuitive and easy to use” = 3). However, he found it rather helpful to complete the required tasks (“Very useful” = 3, “Helped me perform my tasks” = 3).

Regarding the **RUS**, the surgeon responded that the robot had good controls (“Good hand controls” = 4, “Good foot consoles” = 4), was using it comfortably (“Physical comfort” = 4, “NOT stressed/annoyed by instrument” = 3), and it largely worked as intended (“Worked smoothly” = 3, “Worked as wanted” = 3). As expected, however, the 3D vision component was inadequate (“Good 3D vision” = 1) along with the perception that it can be used in real surgeries (“Perform real operation” = 1).

For the evaluation of the **Video**, the image was generally perceived as of high quality (“Colors” = 8, “Contrast” = 8, “Overall quality” = 7), with moderate texture detail (“Textures” = 5) and an absence of 3D (“3D (relief)” = 2). The last point was also mentioned in the free text question from the surgeon, where he responded that the main two difficulties he faced were the lack of depth perception and the latency.

## Discussion

All the exercises were carried out successfully but the lack of depth perception, since the video was not stereoscopic, was a limiting factor. The surgeon’s extensive experience in laparoscopic surgery managed to compensate for this, something which he also noted himself in the free text question. However, the execution speed was reduced. This was largely expected since the use of stereoscopy in robotic surgery has been shown to provide a significant advantage over monocular vision, improving the spatial perception, task efficiency and completion times [37].

Regarding the robotic system, the surgeon managed to get acquainted with it fairly quickly and operated the controls with proficiency. By observing the m-SUS score, the system’s usefulness and usability were viewed in a largely neutral to positive light, something which reflects the need to evolve the robot’s operation and performance. For comparison, the m-SUS reported in [36] for a similar telesurgical experiment using a novel surgical robot was 21.31 ± 4.50 (vs. 28 in our tests) for a 1Gbps transmission line. However, the scores are not directly comparable since there is only one test subject in our trials, and are thus presented for a sense of reference.

Analyzing the items in the RUS, we see that the system was seen largely favorably. Notable exceptions are the perceived unsuitability of the platform for real surgical operations and the system’s lack of 3D vision. The former is expected since the platform is a prototype meant for research and was not built for actual surgery. For the latter, the lack of 3D vision might explain the moderate score for the “texture” rendering in the Video Quality Assessment questionnaire, as the surgeon is used to viewing the surgical field through the high- definition stereoscopic imaging system of a commercial platform. Therefore, reverting to monocular vision might reduce the perceived details of the operating field.

**Fig. 5:**
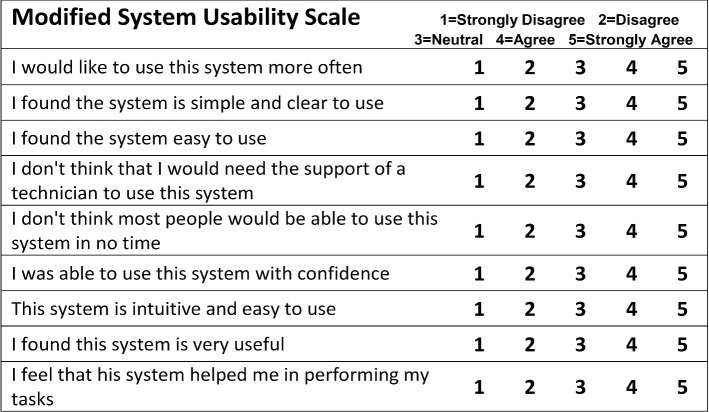
Modified System Usability Scale questionnaire

The implicit comparison with the dominant surgical platform can also explain the reported difficulty with the system’s latency. The measured delay of 350 ms is considered safe but tiring [19] and deviates from what the surgeon is accustomed to. This latency in the imaging pipeline could be further reduced by implementing more suitable streaming architectures for real time communications e.g., peer-to-peer connections (not through a server), appropriate (ultra) low latency video streaming protocols like webRTC and Real Time Streaming Protocol (RTSP), high frame-rate cameras and monitors, etc. The current imaging configuration was “production-grade” and was adopted because of the need for broadcasting the event outside.

## Conclusion

This paper reports on a set of telesurgery experiments over 5 G in Greece, using a novel prototype surgical robot called the “Double Delta.” The experiments consisted of simple surgical exercises on a robotic surgery training kit, and the surgeon was located 300 km away. The round trip delay for the transmission of the manipulation commands was measured to 18 ms while the latency of the video feed was 350 ms. All exercises were performed successfully, with the experience of the surgeon compensating for various limitations of the setup. The results of this study highlight the potential of 5 G technology to significantly enhance the capabilities of telesurgery by providing faster speeds and lower latency which is crucial for real-time data transmission. Future work will focus on evolving the Double Delta platform, enhancing its usability, integrating 3D vision and streaming it in real time over the internet, as well as investigating novel telesurgical concepts such as haptic-enabled telesurgery over 5 G, portable/mobile telesurgery, collaborative telesurgery and others.

**Fig. 6:**
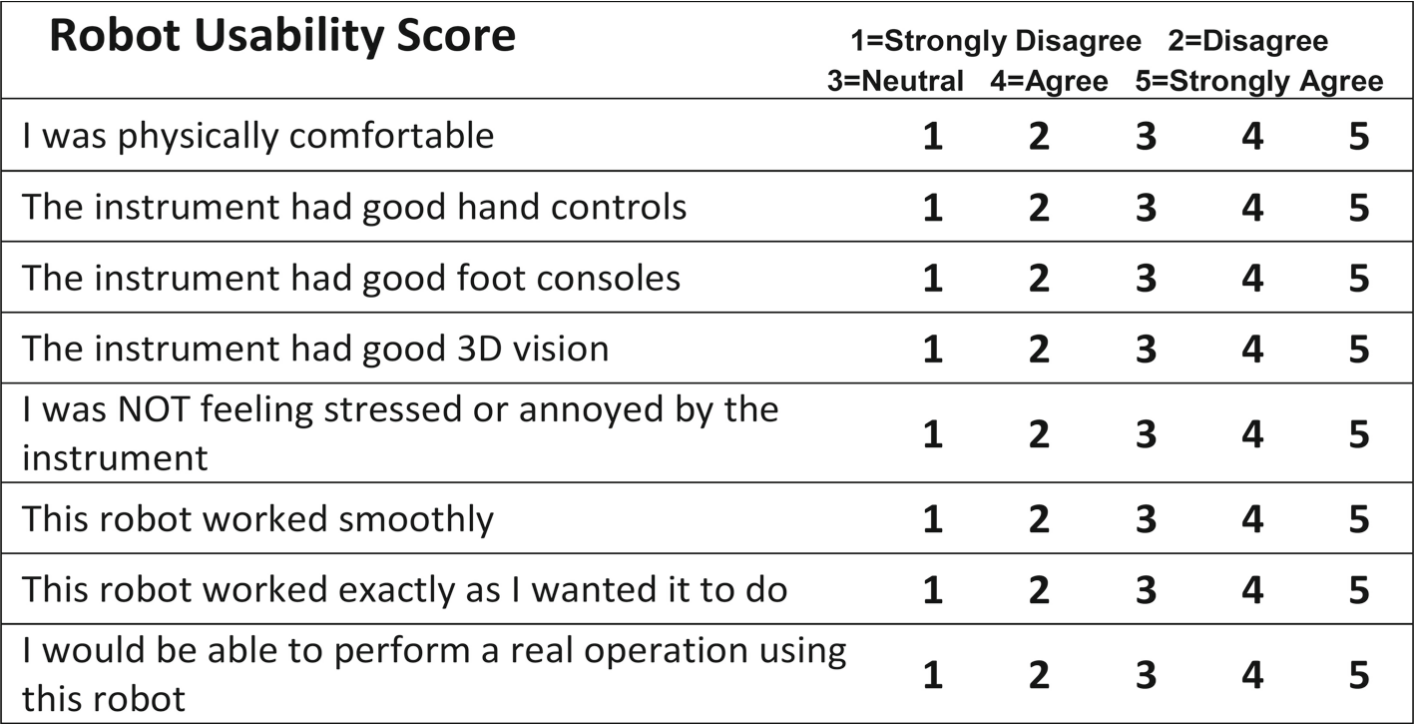
Robot Usability Score questionnaire

**Fig. 7:**
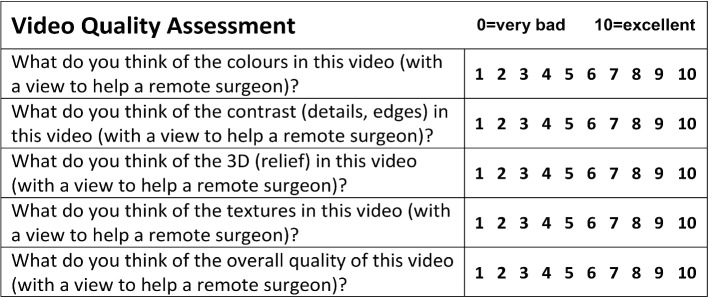
Video Quality Assessment questionnaire

## Supplementary information

Segments of various tasks are presented in the accompanying video.

### Supplementary Information

Below is the link to the electronic supplementary material.

Supplementary file 1 (mp4 16901 KB)

## Appendix A: Evaluation questionnaires

See Figs. 5, 6 and 7.

## Appendix B: List of abbreviations

See Table 1.

**Table 1:** List of abbreviations

Abbreviation	Definition
UL	Upload
DL	Download
CPE	Customer premises equipment
ETH	Ethernet
ms	Millisecond
km	Kilometer
Kbps	Kilobit per second
Mbps	Megabit per second
Gbps	Gigabit per second
FRS	Fundamentals of robotic surgery
UDP	User Datagram Protocol
HD	High definition
OR	Operating room
DoF	Degrees of Freedom
m-SUS	Modified System Usability Scale
RUS	Robot Usability Score
RTSP	Real Time Streaming Protocol
